# Editorial for the *IJMS* Special Issue on “Neurodevelopmental Disorders: From Epigenetic Basis to Therapeutic Perspectives”

**DOI:** 10.3390/ijms25115641

**Published:** 2024-05-22

**Authors:** Miriam Zappella, Roberto Sacco, Alessandra Micera

**Affiliations:** 1 Department of Psychology, Salesian University of Rome, 00139 Rome, Italy; miriamzapp@tiscali.it; 2 Research Unit of Medical Genetics, Department of Medicine, Università Campus Bio-Medico, 00198 Rome, Italy; r.sacco@policlinicocampus.it; 3 Research and Development Laboratory for Biochemical, Molecular and Cellular Applications in Ophthal-Mological Science, IRCCS–Fondazione Bietti, 00184 Rome, Italy

In this Special Issue, we focus on the complex mechanisms underlying neurodevelopmental disorders (as delineated in the DSM-5), which are a group of neurological disorders that begin in childhood but significantly impact adult life. This classification group includes six primary diseases with varying symptoms and deficits, despite sharing the same diagnosis—Intellectual Disability, Communication Disorders, Autism Spectrum Disorder, Attention-Deficit/Hyperactivity Disorder, Neurodevelopmental Motor Disorders and Specific Learning Disorders. These disorders affect one’s personal and social life from childhood to adulthood. Additionally, these neurodevelopmental problems can be associated with structural anomalies of the heart, kidneys, genitourinary tract, ears and eyes. A variety of eye anomalies (mainly optic nerve anomalies and microphthalmia) and vision issues (myopia, anisometropia, astigmatism, exotropia, esotropia) have been reported, with the explanation for these found in the fact that the brain and eyes share the same embryonic origins.

Epigenetic changes, such as DNA methylation and alterations in micro-RNA, have been linked to this group of diseases. Mutated genes also play a role in affecting synaptic transmission and causing dysfunction in plasticity reactions. These genes not only contribute to the pathogenesis of neurodevelopmental disorders but also regulate synaptogenesis and signaling pathways. Various studies have shown that mutated genes are involved in both neurodevelopmental and psychiatric disorders. Understanding the genetic causes of neurodevelopmental disorders is crucial for implementing primary prevention strategies in the future. The stability of some epigenetic marks and their potential to persist into the next generation can lead to the long-term inhibition of gene expression.

Against this backdrop, this Special Issue comprises two original research papers, one communication and two informative review articles, providing comprehensive insights into neurodevelopmental disorders discussed by experts in the field.

Bolognesi et al. [1] considered the 25 kD synaptosomal-associated protein (SNAP-25) a key player in the pathogenesis of Autism Spectrum Disorder (ASD). Focusing on four single nucleotide polymorphisms (SNPs) of the SNAP-25 gene (rs363050, rs363039, rs363043 and rs1051312), the authors found that patients who experienced symptoms in the first life year more frequently had the rs363039 G allele and GG genotype. On the contrary, children who experienced a regression during the second life year more frequently had the rs1051312 T allele and TT genotype. Therefore, the authors hypothesized that these genetic variants could provide indications regarding the timing of symptom manifestation. One possible epigenetic role in the onset of regressive autism could be attributed to rs1051312, as it is involved in microRNA (miRNA) regulation. Since SNAP-25 is essential for pre- and post-synaptic transmission, as well as post-synaptic formation and adhesion (spine morphogenesis), the authors hypothesized that these two SNAP-25 variants could serve as predictive genetic and diagnostic markers.

Krzyzewska et al. [2] argued that in Fetal Alcohol Spectrum Disorder, six differentially methylated regions (annotated to the SEC61G, REEP3, ZNF577, HNRNPF, MSC and SDHAF1 genes) were associated with changes in gene expression. The authors expanded their previously published study by enlarging the FASD cohort to investigate the effects of DNAm on gene expression. A correlation between DNAm changes and gene expression was first demonstrated, and, thereafter, the functional integrative effects of altered DNAm status on gene expression through an expression quantitative trait methylation (eQTM) analysis was examined.

Hu et al. [3] studied the importance of PRKCA in regulating social interactions and social behavior by analyzing a PRKCA-deficient zebrafish model (Danio rerio). The molecular analysis conducted showed that Prkcaa and Prkcab were actively expressed in several tissues, except liver. The highest prkcaa transcript expression was found in the ovaries, followed by the eyes and brain, whereas the highest prkcab transcript expression was found in the brain, followed by the eyes and gills. Both proteins were distributed in the cytoplasm, while Prkcab was also observed at the membrane level. RNA sequencing revealed that dysfunction in PRKCA had a significant impact on the downregulation of a group of circadian genes at night. In zebrafish displaying anxiety-related behaviors and compromised social interactions, there was a deficiency of PRKCA, especially in the morning-preferred circadian gene expression of egr2a, egr4, fosaa, fosab and npas4a, which, during the night, were not repressed due to PRKCA dysfunction. The authors stressed that the day–night locomotor rhythm was inverted, demonstrating the crucial role of PRKCA in regulating social interactions and circadian rhythm. The authors concluded that the protein kinase Cα (PKCα/PRKCA), in particular, plays an important role in the circadian rhythm and in the manifestation of Autism Spectrum Disorder and Schizophrenia.

Sun et al.’s review [4] highlighted how changes in gut microbiota and the metabolic dysregulation of tryptophan metabolites can occur following a traumatic brain injury. The process is worsened by a neuroinflammatory response that, in turn, can be mediated and modulated by the gut–brain–microbiota axis. This review specifically explored the role played by the Aryl hydrocarbon Receptor (AhR), tryptophan and tryptophan co-metabolism in the gut microbiota, and the signal pathway triggered following a traumatic brain injury, indicating potential future targets for intervention.

Fulceri et al.’s review [5] delved into and identified existing care pathways from referral to post-diagnosis management for individuals with autism through strict criteria in published articles. They examined the challenging transition from adolescence to adulthood, finding that what emerges is the need for multidisciplinary care and effective coordination among various healthcare/social service providers.

Overall, given the exponential increase in neurodevelopmental disorders in recent years, it is essential to stress that delving deeper into etiopathogenesis factors, and particularly epigenetic targets, might reinforce the contributions of classic biomolecular determinants.
